# Supplementary material for: Reliability and validity of neurobehavioral function on the Psychology Experimental Building Language test battery in young adults
Source: PeerJ. 2015 Dec 22;3:e1460. doi: 10.7717/peerj.1460 (PMC4690381; doi:10.7717/peerj.1460)
Supplement: Tables S1--S3 [file peerj-03-1460-s002.pdf]

Supplemental Table 1. Selected peer-reviewed publications using the Psychology Experiment Building Language (PEBL) software. Berg/Wisconsin Card Sorting Test (BCST); Delayed Match to Sample (DMS); Implicit Association Task (IAT); Iowa Gambling Task (IGT); Psychomotor Vigilance Task (PVT); Situation Awareness Task (SAT); Time-Wall (TW); Tower of London (ToL); and Trail Making Test (TMT).

Topic	1st Author	Year	Citation	PEBL Test(s)
Description of PEBL	Mueller	2010	*International J of Machine Consciousness* **2**: 273-288	TMT, IGT, many others
Pursuit rotor in children	Piper	2010	*J Neuroscience Methods* **195**:88-91	Pursuit Rotor
Alcohol & decision making	Lyvers	2010	*Addictive Behaviors* **35**: 1021-1028	BCST
Anxiety & decision making	de Visser	2010	*Neuropsycholia* **48**:1598-1606	BCST
Caffeine & decision making	Aggarwal	2011	*British J Surgery* **98**: 1666-1672	Stroop, BCST, PVT
Behavioral genetics of glutamate	Ness	2011	*Neuropharmacology* **61**:950-956	IGT
Heavy drinkers & decision making	Gullo	2011	*Drug & Alcohol Dependence* **117**:204-210	Digit span
Behavioral genetics & amphetamine	Wardle	2012	*Genes, Brain & Behavior* **12**:13-20	BCST, N-back
Schizotypy & cognition	Cappe	2012	*Psychiatry Research* **200**:652-659	BCST
Brain damage & strategy updating	Danckert	2012	*Cerebral Cortex* **22**:2745-2760	BCST
Multiple sclerosis and cognition	Kalinowska	2012	*J of Neurological Sciences* **321**: 43-48	reaction-time
Executive function & lifespan	Piper	2012	*Behavior Research Methods* **44**: 110-123	BCST, TMT, ToL, TW
Aging & executive function	Zebrowitz	2013	*Psychology & Aging* ***28***: 202-212	BCST
Transcranial Infrared Laser	Barrett	2013	*Neuroscience* **230**: 13-23	PVT, DMS

Obsessive Compulsive Disorder	Tumkaya	2013	*Psychiatry Research* **209**: 579-588	SAT
Wilson's disease & decisions	Ma	2013	*J Clin Exp Neuropsychol* **35**: 472-479	BCST
Alcohol consumption & decisions	Bowley	2013	*Int J Psychophysiology* **89**: 342-348	IAT
Essential tremor & cognition	Bhalsing	2014	*Eur J Neurology* **21**; 874-883	BCST
Tourette's & motor skill	Brandt	2014	*PLoS One* **9**: e98417	Pursuit Rotor
Executive function & Parkinson's	Cohen	2014	*J Parkinson's Dis* **4**: 111-122	BCST
Hoarders and cognition	Raines	2014	*J Affect Dis* **166**: 30-35	CPT
Sex differences	Evans	2015	Brain & Cogn **93**: 42-53	IGT
Decision making & alcohol	Lyvers	2015	*Addict Behav* **41**: 129-135	IGT

* For an updated list, see: http://pebl.sourceforge.net/wiki/index.php/Publications_citing_PEBL

Supplemental Table 2. Comparison of the antecedents to measures contained in the Psychology Experiment Building Language (PEBL) battery including the originator(s), year of key publication, construct measured, and title for PEBL version. Berg Card Sorting Test (BCST); Executive Function (EF); Iowa Gambling Task (IGT); Mental Rotation Test (MRT); Tower of London (ToL); Test Attentional Vigilance (TOAV); Trail Making Test (TMT)

Test	Originator	Year	Construct	PEBL Version
Digit Span	Alfred Binet	1905	working memory	Digit Span
Rotary Pursuit	Robert Ammons	1947	procedural learning	Pursuit Rotor
Wisconsin Card Sorting Test	David Grant; Esta Berg	1948	EF: set shifting	BCST
TMT	Ralph Reitan	1955	EF: divided attention	TMT
MRT	Roger Shepard	1978	EF: decision making	MRT
ToL	Tim Shallice	1983	EF: planning	ToL
Test of Variables of Attention	Lawrence Greenberg	1993	sustained attention	TOAV
IGT	Antoine Bechara	1994	decision making	IGT

Supplemental Table 3. Mean performance and correlation between test sessions on Psychology Experiment Building Language measures. The number of participants is listed in ( ) after each test; [r]reported previously[47]; Coded according to the [O]oringal Berg sorting rules or the [H]Heaton rules; [A]t-test $P \leq .01$ versus test; [B]correlation $P < .01$.

	Test		Retest		%	Cohen's	Correlation	
	Mean	SEM	Mean	SEM	Difference	d	Pearson r	rho
Rotary Pursuit (76)								
Total time (sec)	37.9	1.1	43.7[A]	0.9	+15.4	.60	.86[B]	.81[B]
Error (pixels)	31.7	3.3	24.7	2.7	-22.0	.24	.59[B]	.77[B]
Trail Making Test (78)								
Time A (sec)[r]	16.5	0.3	15.4[A]	0.3	-6.6	.07	.74[B]	.71[B]
Time B (sec)[r]	22.1	0.5	19.3[A]	0.5	-12.8	.13	.61[B]	.56[B]
Ratio (B/A)	1.35	0.03	1.25[A]	0.02	-7.2	.07	.39[B]	.35[B]
Digit Span Forward (72)	12.2	0.48	13.0	0.44	+6.6	.20	.63[B]	.62[B]
Test of Attentional Vigilance (68)								
Response Time	385.4	4.8	395.2	7.7	+2.5	.25	.79[B]	.72[B]
SD of Response Time	109.8	5.3	98.9[A]	6.7	-9.9	.25	.87[B]	.69[B]
Omission Errors	8.1	1.9	5.7	1.4	-30.2	.16	.88[B]	.43[B]
Commission Errors	16.9	1.1	13.8	1.3	-18.3	.34	.65[B]	.66[B]
Tower of London (66)								
Moves	8.9	0.2	9.0	0.3	+1.3	.09	.15	.34[B]
Time (sec)	16.8	0.5	14.1[A]	0.5	-16.0	.62	.36[B]	.48[B]
Iowa Gambling Task (68)								
Response pattern	8.8	2.9	18.2[A]	3.5	+106.3	.39	.41[B]	.22
Money	1944.8	85.0	2162.1	116.0	+11.2	.31	.10	-.01
Berg Card Sorting Test (73[O]/60[H])								
Categories Completed[O]	3.1	0.2	3.6[A]	0.2	+16.0	.39	.51[B]	.47[B]
Errors (%)[O]	30.0	1.7	22.0[A]	1.6	-26.7	.55	.69[B]	.68[B]
Perseverative responses[O] (%)	30.7	0.9	31.1	1.0	-1.3	.05	.03	.00
Perseverative errors[O] (%)	14.9	0.8	12.2[A]	0.9	-18.2	.41	.45[B]	.35[B]

Perseverative responses[H] (%)	14.5	1.1	12.4	1.4	-24.9	.14	.51[B]	.35[B]
Perseverative errors[H] (%)	13.0	0.9	11.2	1.0	-27.7	.14	.53[B]	.34[B]
Time-Wall (74)	.175	.02	.070[A]	.006	-60.0	.68	.39[B]	.61[B]
