# Supplementary material for: Reliability and validity of neurobehavioral function on the Psychology Experimental Building Language test battery in young adults
Source: PeerJ. 2015 Dec 22;3:e1460. doi: 10.7717/peerj.1460 (PMC4690381; doi:10.7717/peerj.1460)

Supplementary Figure 1. Psychology Experiment Building Language (PEBL) screen-shots including Pursuit Rotor (A), Time-Wall (B), Trail-Making Test (C), Digit Span (D), Wisconsin (Berg) Card Sorting Test (E), Mental Rotation (F), Tower of London (G), Dexterity (H), and the Test of Attentional Vigilance (I). The source code (*.pbl files) is also at: https://github.com/stmueller/pebl-custom

A) Pursuit Rotor, a test of fine-motor learning, instructions (top) and example trial (bottom).

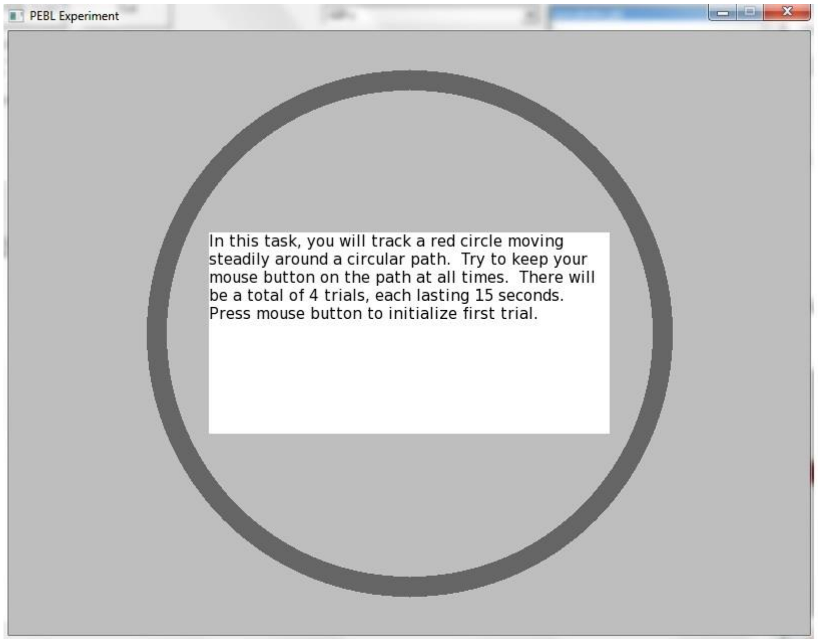

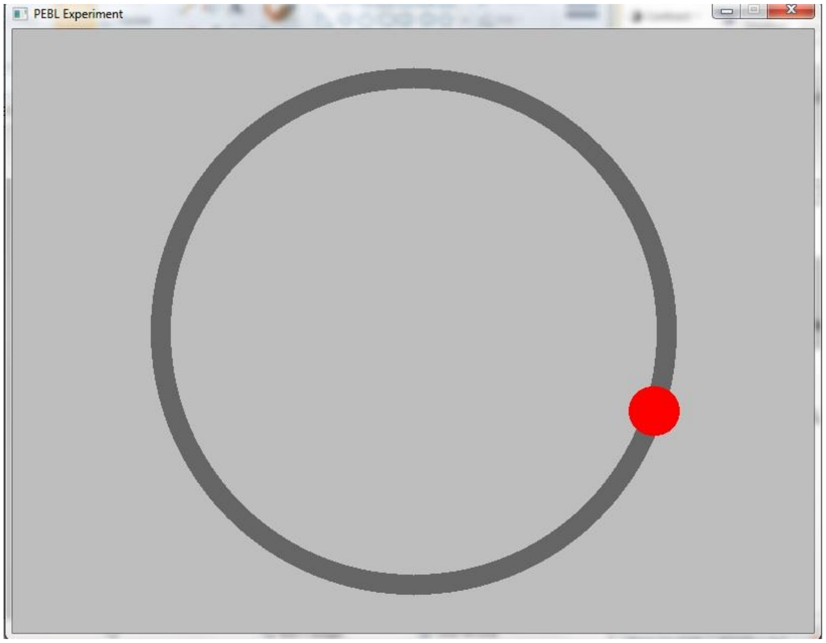
PEBL Experiment

B. Time-Wall, an index of attention and decision-making, instructions (top) and trial (bottom).

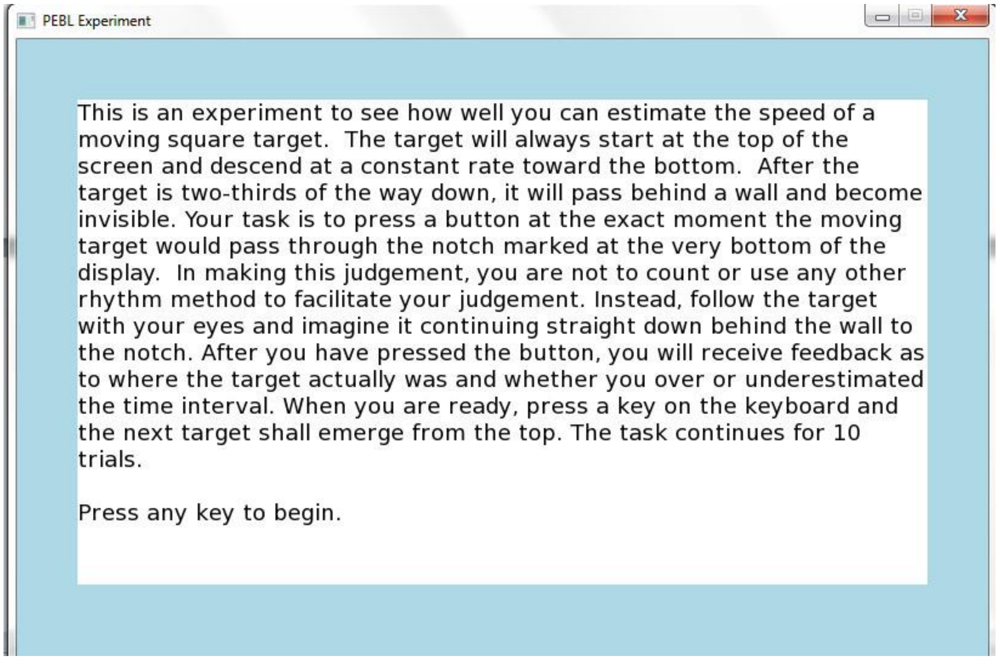

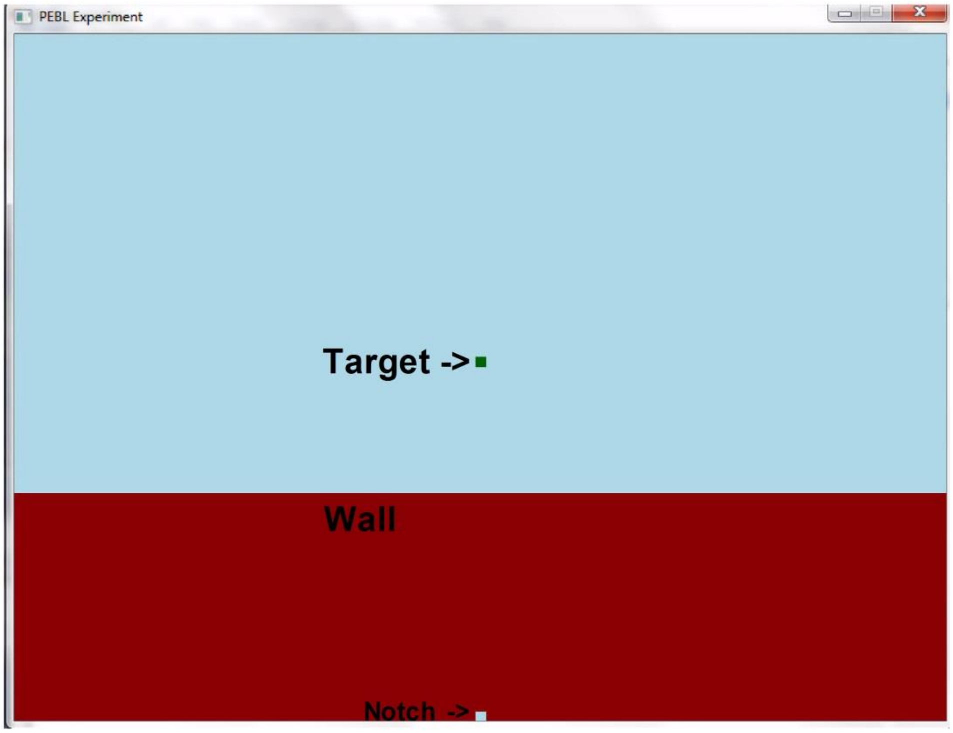

C. PEBL Trail-Making Test, a measure of executive function (set-shifting), instructions (top) and example B trial (bottom).

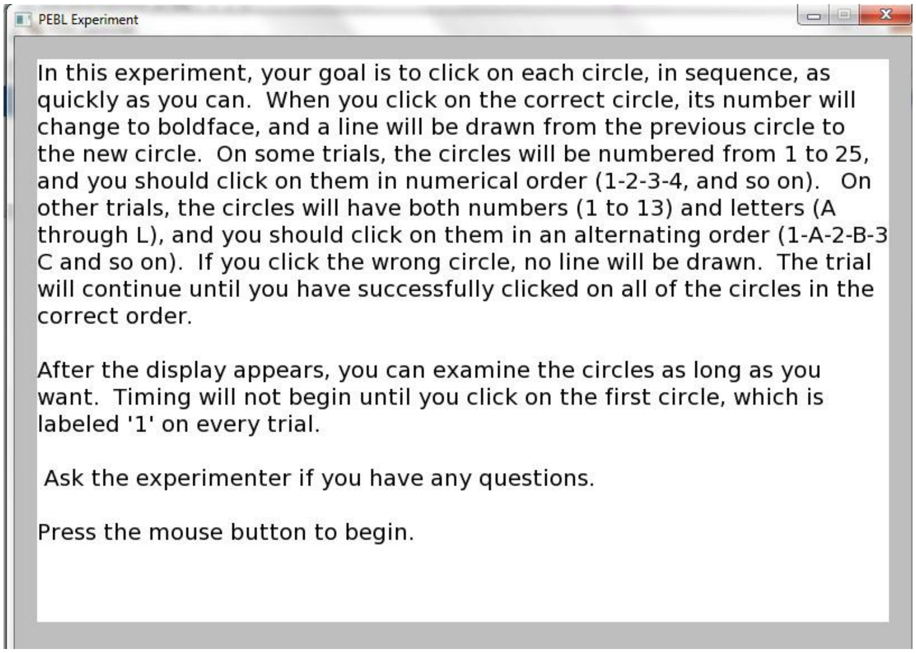

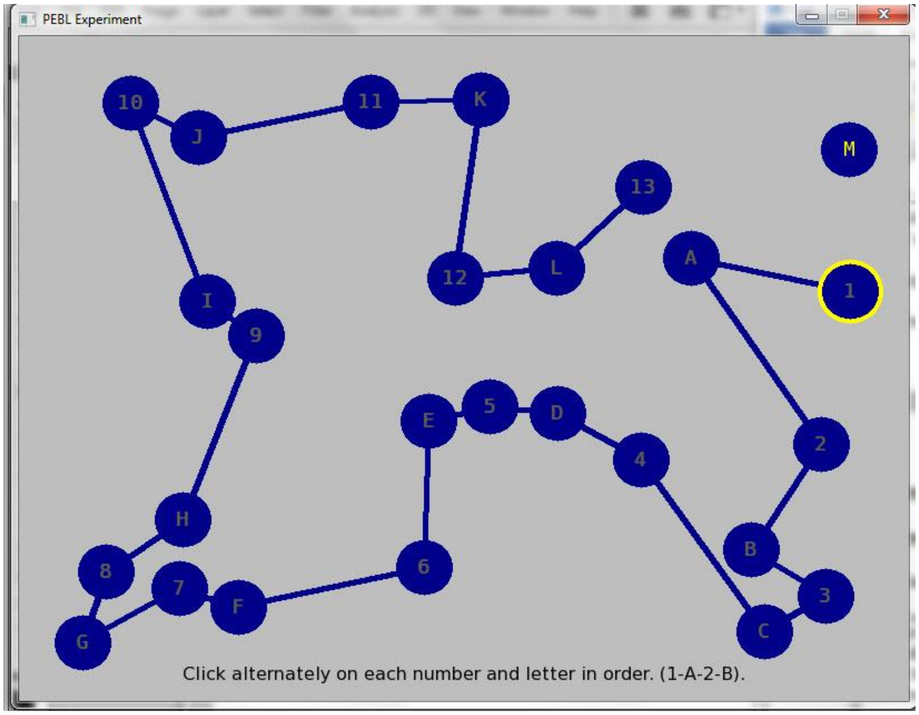

D. PEBL Digit Span, a measure of working memory, instructions.

You are about to take part in a memory test. You will be presented with a sequence of digits, one at a time on the screen.Each digit will occur only once during a list. You will then be asked to type the list of digits exactly in order. If you do not know what digit comes next, you can skip over it by typing the '-' key. Once entered, you cannot go back to edit your responses. You will start with a list of three items, and will get three different lists at each length. If you are able to recall two out of three lists completely correctly, you will move on to the next longest list length.

E. PEBL Berg Card Sort Test, a test of executive function (set shifting), instructions (top and middle) and example trial with color as the current rule (bottom).

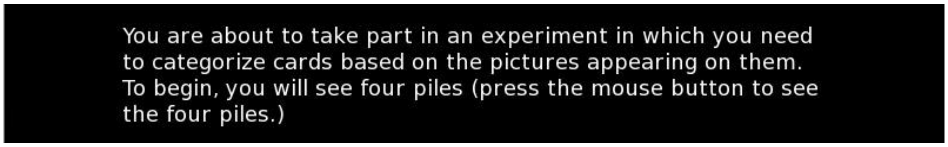

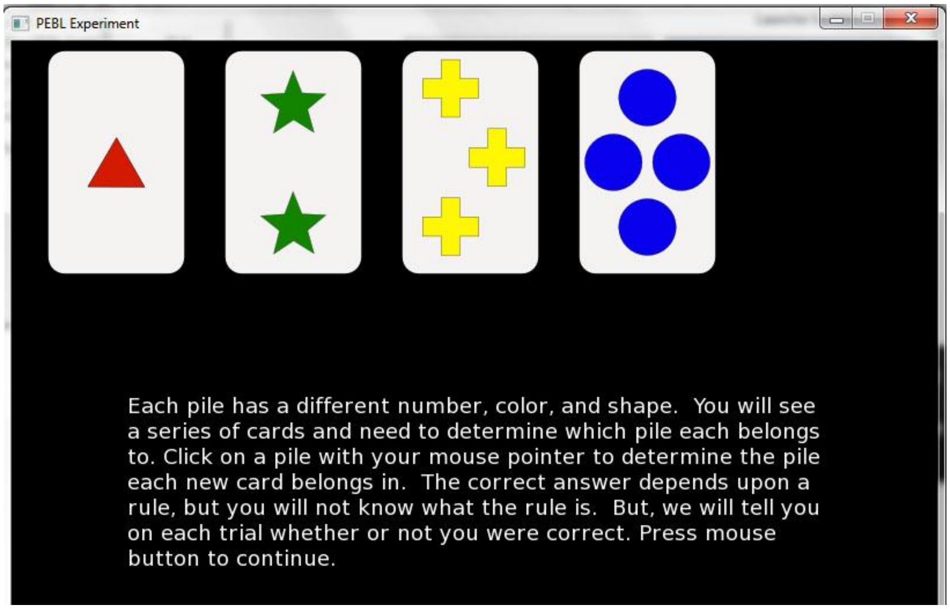

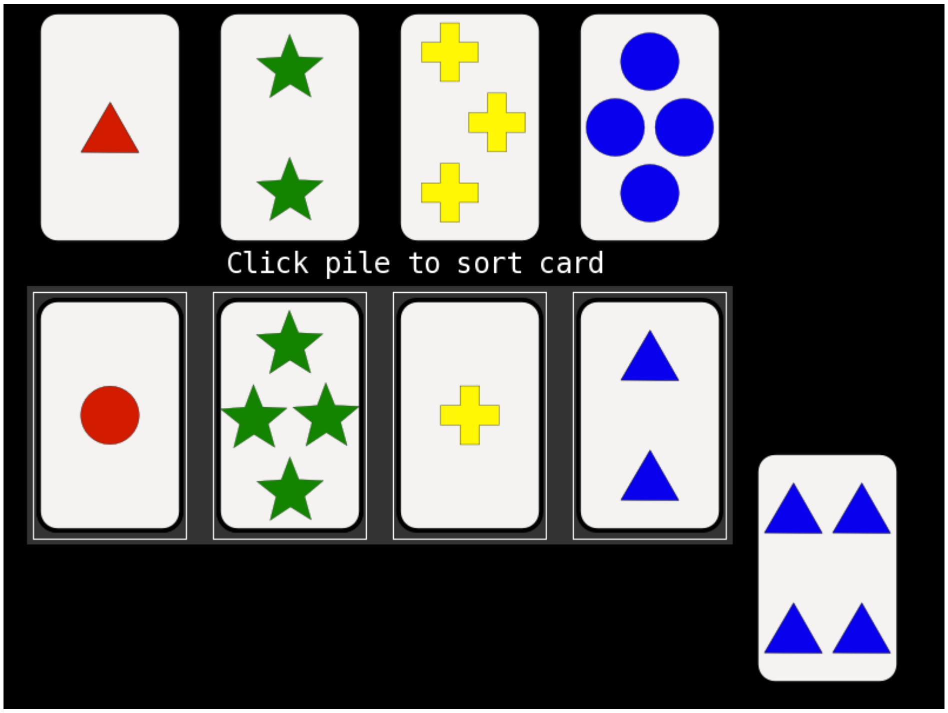

F. Mental Rotation, an index of executive functioning, instructions (top) and example stimuli (bottom).

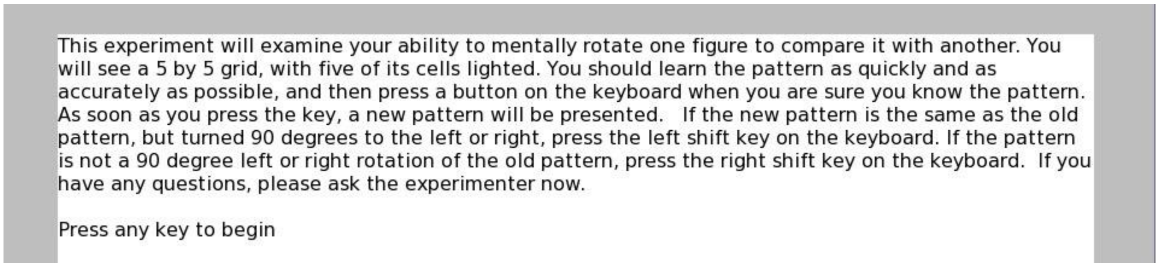

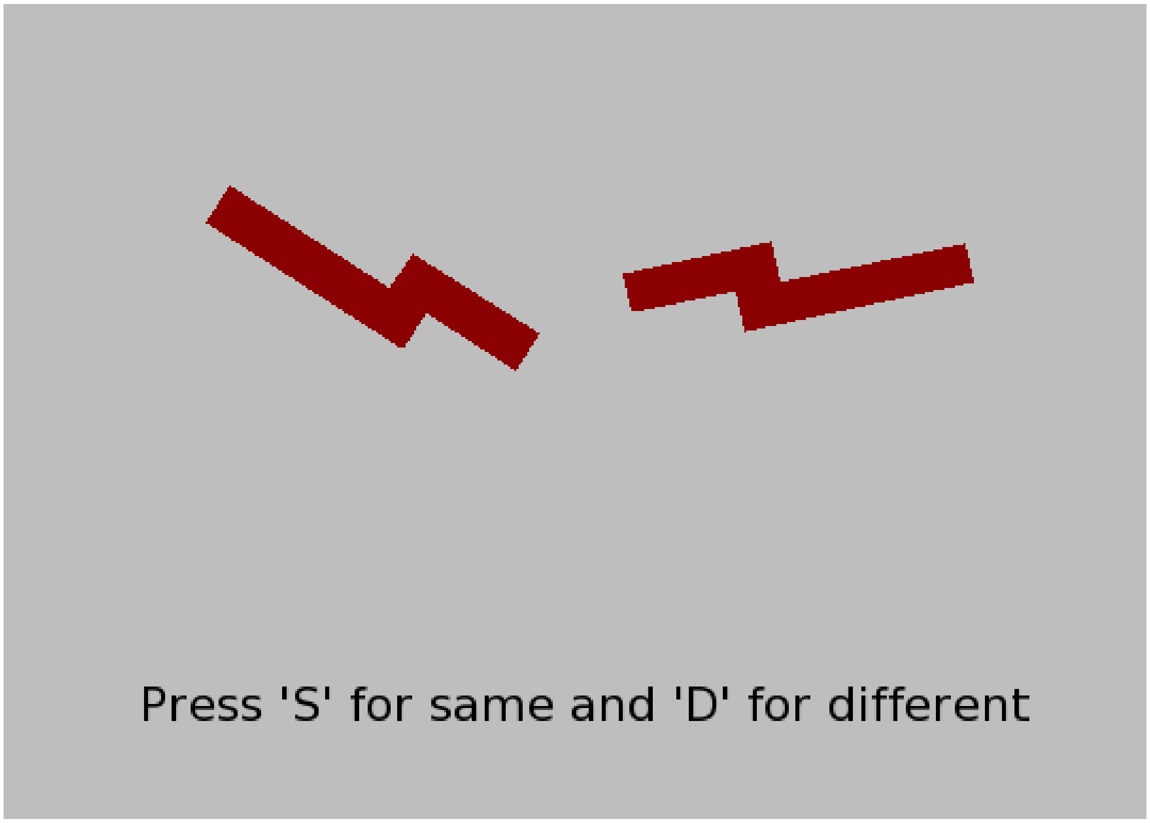

G. PEBL Tower of London, a measure of executive-function (planning), instructions (top) and a sample trial (bottom). This version was used in Study II.

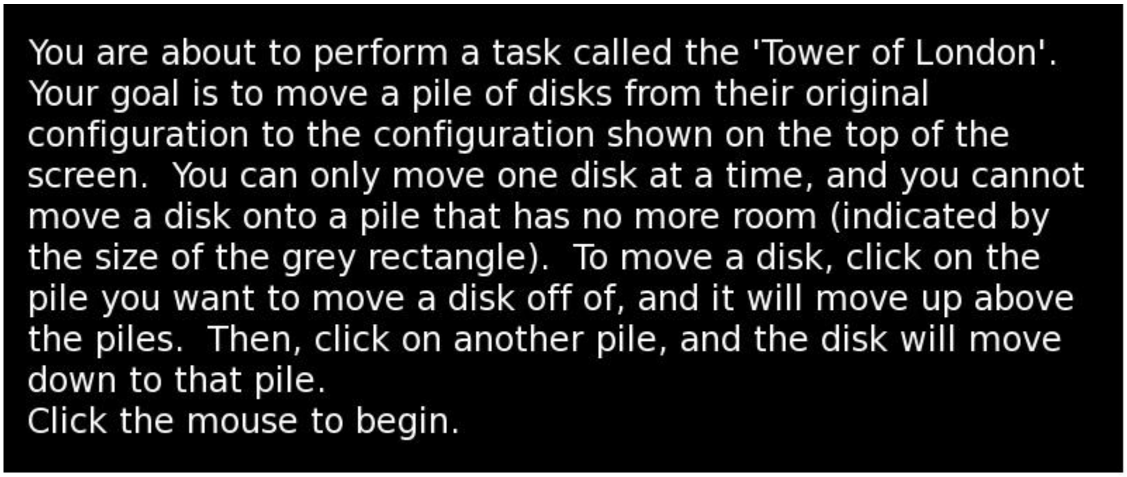

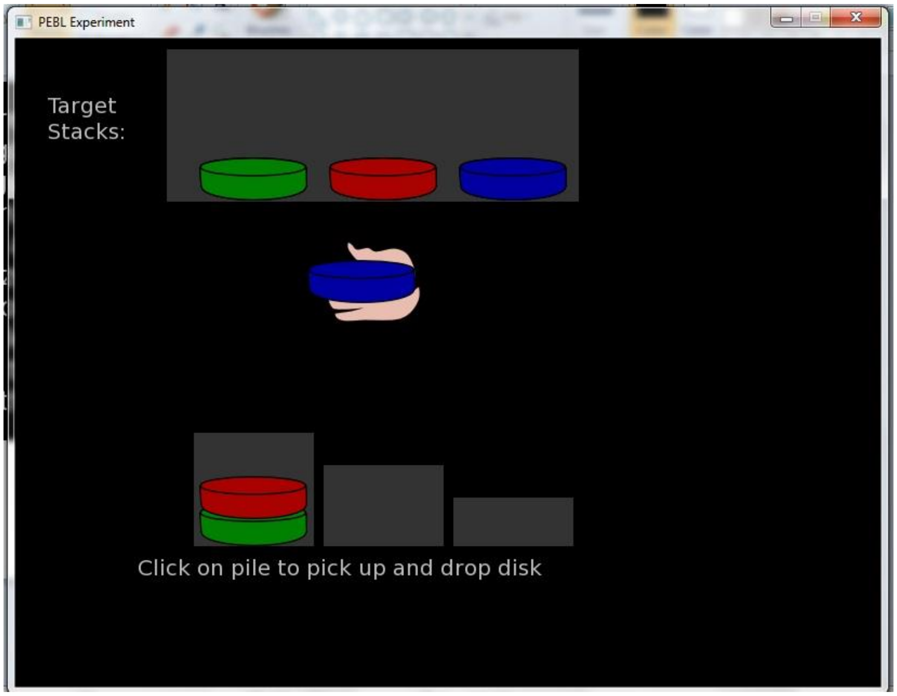

H. Dexterity, a test of fine motor ability, instructions (top) and example trial (bottom). Dexterity is a recently developed test of fine motor function which consists of a circular coordinate plane with the center of the circle (demarcated by a thin black line) at x,y positions 0,0. The goal is to move the cursor (depicted as a colored ball) to a target located at various positions. Movement of the cursor is affected by a "noise" component complementing the directional input from the analog mouse to create the effect of interference or "jittering" motion. The effect is such that successful navigation of the coordinate plane using the mouse encounters resistance to purposeful direction, requiring continual adjustment by the participant to maintain the correct path to the target. Visual feedback is given by the use of a color system, wherein the cursor shifts gradually from green to red as proximity to the target becomes lesser. The task consists of 80 trials (10 per "noise" condition), ten seconds maximum in length, with preset noise factors (ranging in intensity) and target locations standardized for consistency between participants. A lack of input from the participant results in a gradual drift towards the center. At the conclusion of each trial, the cursor location is reset to the origin. Completion time and Moves were recorded with Moves defined as the change in the vector direction of the mouse while course correcting toward the target. The radius of the circular coordinate plane is defined as a function of the screen size and resolution as 300 arbitrary units, the cursor as 2.5 units, and the target as 12.5 units. Distance to the target is computed using those arbitrary units. Cursor velocity is defined externally as "intermediate" in the Windows XP Service Pack 2 settings.

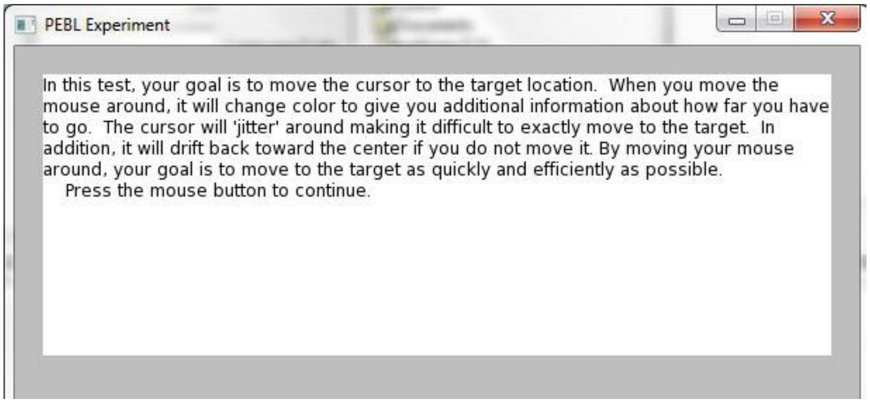

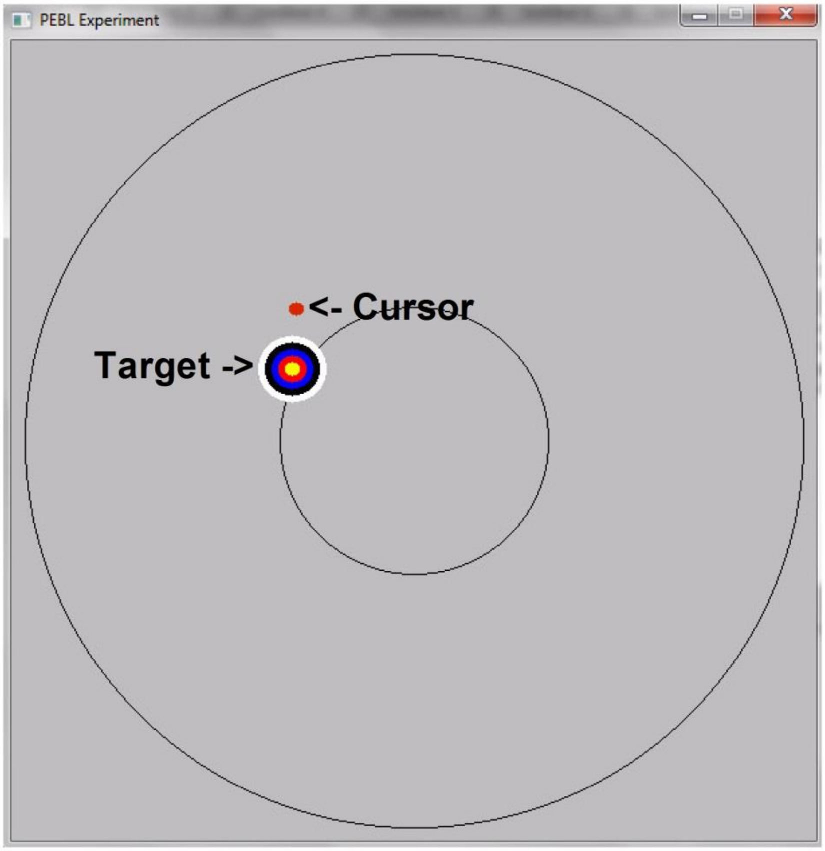
PEBL Experiment
<- Cursor
Target ->

I. Test of Attentional Vigilance (TOAV), a measure of sustained attention, example trial. The instructions were as follows:

On each trial, you will see one of two stimuli on the screen. Each will be a white square with a black square inside it. On some trials, this inner square will be near the top of the white square; on other trials it will be near the bottom. Press any key to see the stimuli.

When the square is on the top, it is a target. During the task, you should press the space bar whenever you see the target stimulus. Press any key to continue.

When the square is on the bottom, it is NOT a target. During the task, you should not press the space bar when the non-target is displayed. Press any key to continue.

During the task, you will see a series of targets and non-targets. Press the space bar as quickly as you can whenever you see a target (top square). Do nothing when you see a non-target (bottom square). The task lasts approximately 6 minutes, so you need to concentrate on the task in order to perform well. Press the space bar to begin.

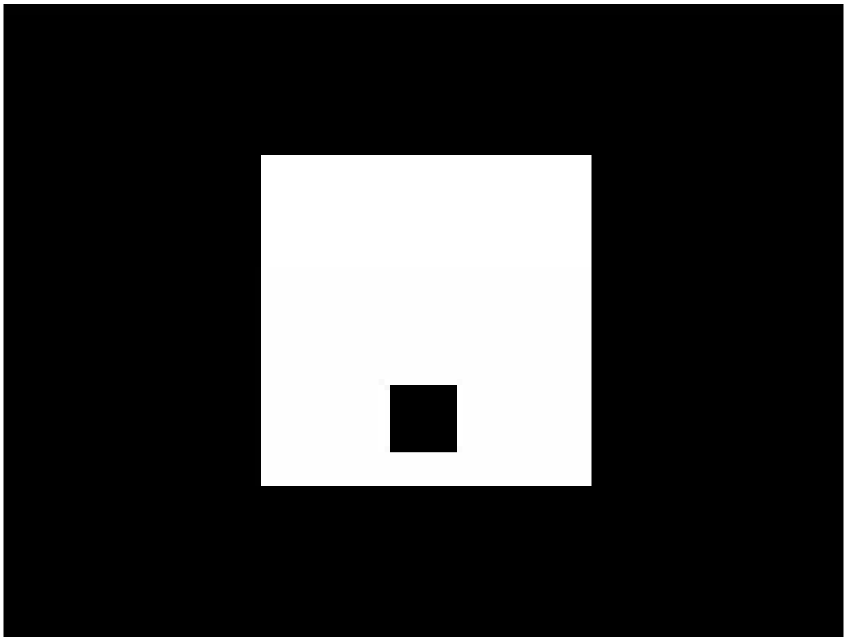

Supplement: Figure S1 [file peerj-03-1460-s003.pdf]
